# Supplementary material for: Exploration of key mechanisms underlying the therapeutic effects of AMD3100 on attenuating lipopolysaccharide-induced acute lung injury in mice
Source: PeerJ. 2024 Dec 12;12:e18698. doi: 10.7717/peerj.18698 (PMC11646417; doi:10.7717/peerj.18698)
Supplement: Supplemental Information 1 [file peerj-12-18698-s001.docx]

Exploration of key mechanisms underlying the therapeutic effects of AMD3100 on attenuating lipopolysaccharide-induced acute lung injury

Zhou Lv#1, Bohan Zhang#1, Hui Zhang1, Yanfei Mao1, Qihong Yu*2, Wenwen Dong*1

* Correspondence: Wenwen Dong, dongwenwen@xinhuamed.com.cn;

Qihong Yu, yuqihongaaaa@163.com.

1 Department of Anesthesiology and Surgical Intensive Care Unit, Xinhua Hospital, Shanghai Jiaotong University School of Medicine, Shanghai 200092, China

2 Department of Gastroenterology, Changhai Hospital, The Second Military Medical University, Shanghai 200433, China.

# Zhou Lv and Bohan Zhang contributed equally to this study.

The raw data of our study has been uploaded to NCBI SRA, and could be searched in NCBI.The accession number is SRR28351781,SRR28351780,SRR28351779,SRR28351778,SRR28351777

SRR28351776,SRR28351775,SRR28351774,SRR28351773,SRR28351772,SRR28351771,SRR28351770,SRR28351769,SRR28351768,SRR28351767,SRR28351766.

The number of our BioProject is PRJNA1085512.

The number of our BioSample isSAMN40354775.
