# Supplementary material for: Exploration of key mechanisms underlying the therapeutic effects of AMD3100 on attenuating lipopolysaccharide-induced acute lung injury in mice
Source: PeerJ. 2024 Dec 12;12:e18698. doi: 10.7717/peerj.18698 (PMC11646417; doi:10.7717/peerj.18698)
Supplement: Supplemental Information 3 [file peerj-12-18698-s003.docx]

**The raw numerical data for Figure 1**

| **Histopathologic Damage Score** | | | | | | | |
| --- | --- | --- | --- | --- | --- | --- | --- |
| **Control** | | **LPS** | | **Control+AMD3100** | | **LPS+AMD3100** | |
| Pathologist1 | Pathologist2 | Pathologist1 | Pathologist2 | Pathologist1 | Pathologist2 | Pathologist1 | Pathologist2 |
| 0 | 0 | 4 | 4 | 0 | 0 | 2 | 2.5 |
| 1 | 1 | 4 | 3.5 | 1 | 0.5 | 1 | 1.5 |
| 1 | 0.5 | 5 | 4.5 | 0 | 0.5 | 3 | 2.5 |
| 1 | 1 | 3 | 3 | 1 | 1 | 2 | 1.5 |
| 0 | 0 | 3 | 3.5 | 2 | 2 | 2 | 2 |
| 1 | 1.5 | 5 | 4.5 | 0 | 0 | 2 | 1.5 |
| 1 | 0.5 | 3 | 3 | 1 | 1 | 1 | 0.5 |

**Hematoxylin and eosin staining**

**The** **full lung image**

**The Control group**

**
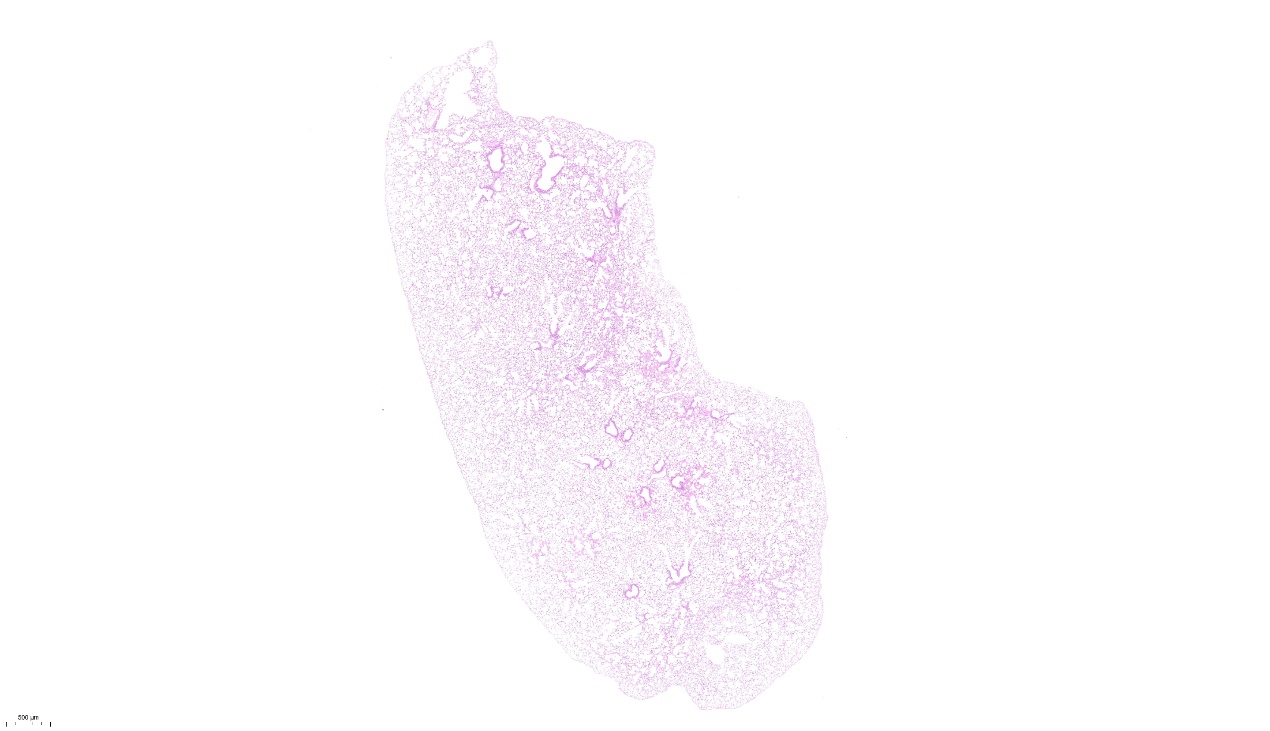
**

**The LPS group**

**
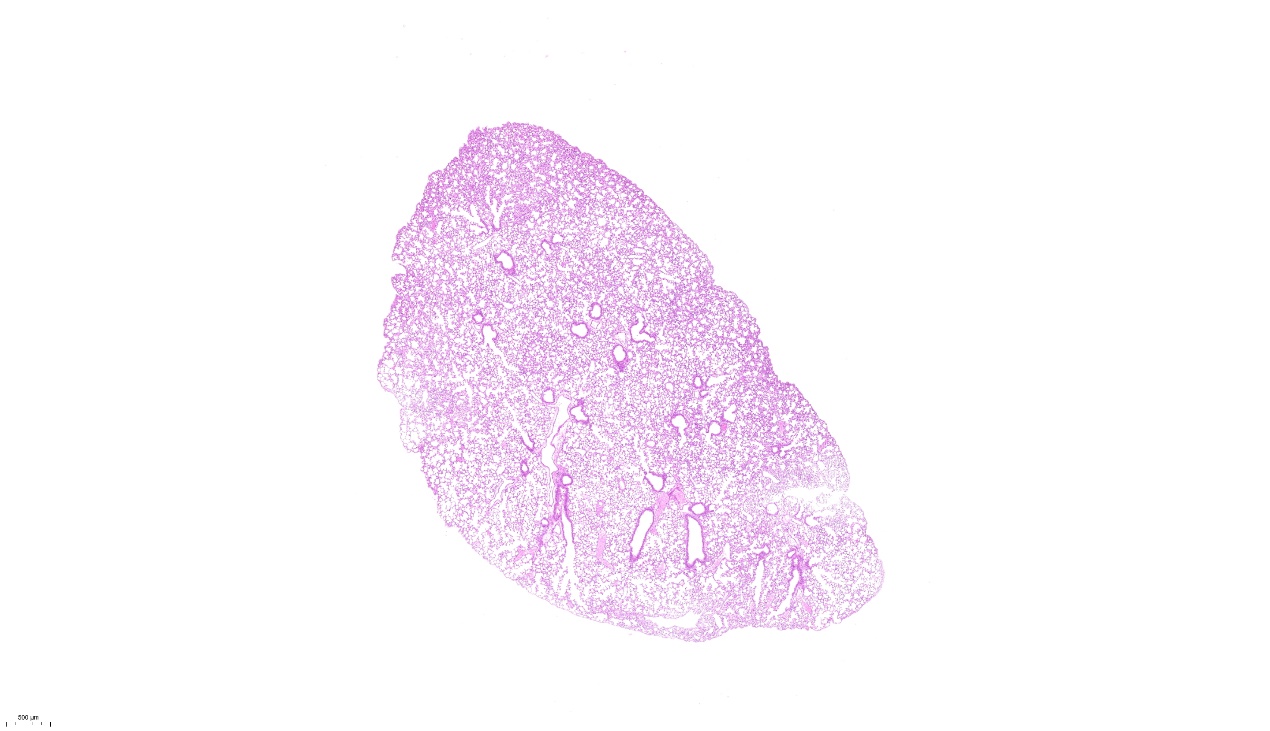
**

**The AMD group**

**
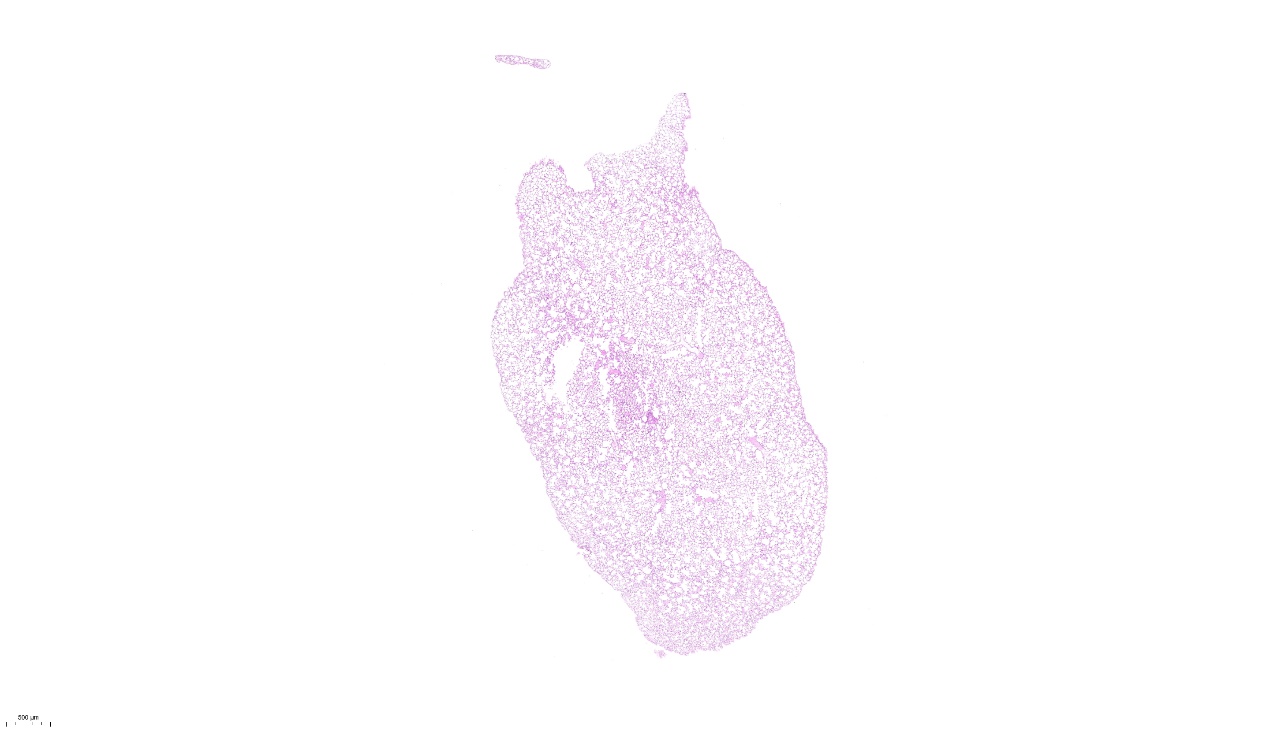
**

**The LPS+AMD group**

**
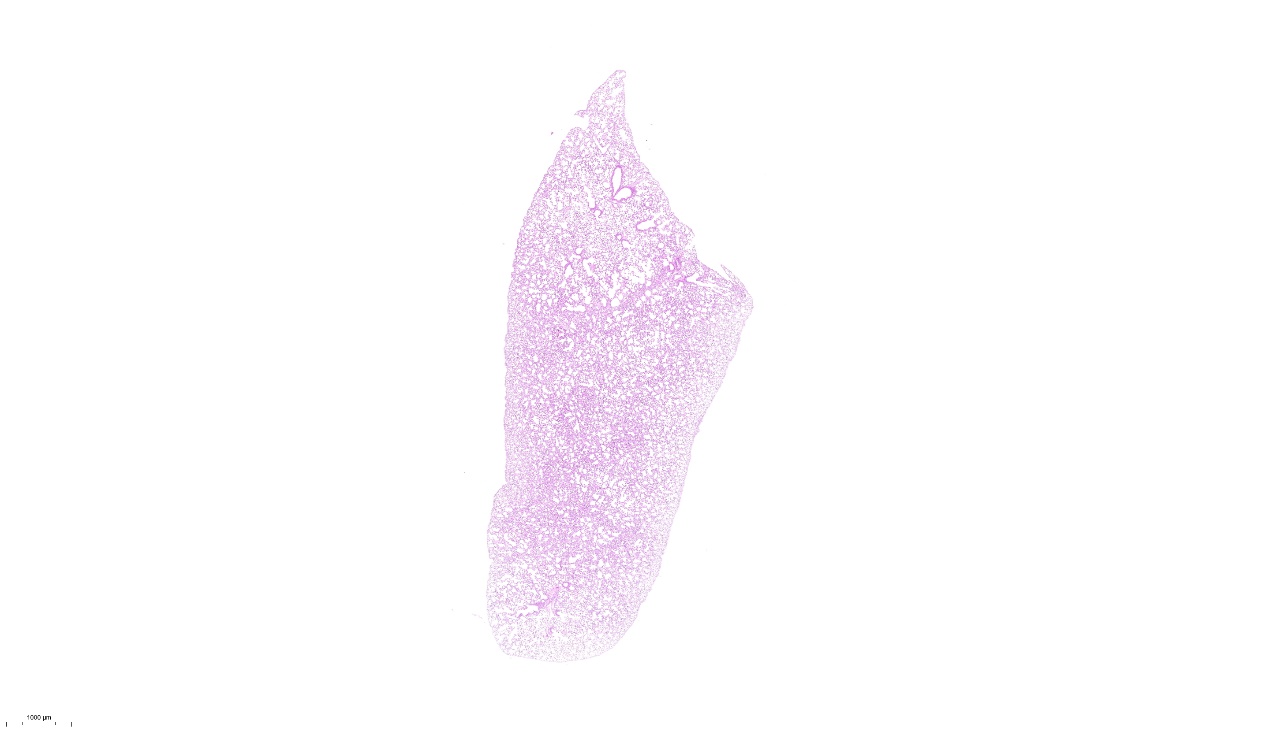
**
